# Guiding significance of the autophagy intensity of lumbar intervertebral discs and the Charlson Comorbidity Index in predicting the postoperative curative effect of patients with single-level lumbar disc herniation

**DOI:** 10.1038/s41598-025-03539-x

**Published:** 2025-06-04

**Authors:** Zhipeng Yao, Taotao Lin, Rongcan Wu, Hailin Lin, Xianfeng Lin, Chao Qin, Tengbin Shi, Xiaoqing Ye, Linquan Zhou, Gang Chen, Zhenyu Wang, Wenge Liu

**Affiliations:** 1 Department of Orthopedics, Fujian Medical University Union Hospital, Fuzhou, 086-350001 China; 2 Fujian Medical University, Fuzhou, 086-350001 China

**Keywords:** Lumbar disc herniation, Autophagy intensity, Charlson Comorbidity Index, JOA improvement rate, Diseases of the nervous system, Spine regulation and structure

## Abstract

To explore whether the autophagy intensity of lumbar intervertebral discs or Charlson Comorbidity Index(CCI) can predict the postoperative curative effect of single-level lumbar disc herniation(LDH) in patients. According to age stratification, five patients with single-level LDH who underwent surgical treatment in our hospital were included in each age group, and the autophagy level of the resected lumbar disc was detected by immunohistochemistry. A total of 30 patients were included and followed up for 2 years. According to the JOA improvement rate at the last follow-up, the patients were divided into two groups. According to age stratification, we found that there were significant differences in autophagy intensity and Pfirrmann classification; that is, with older age, the degree of lumbar disc degeneration was more serious and the autophagy intensity was lower. According to the JOA improvement rate, we found that there were significant differences in age, Autophagy intensity, Pfirrmann classification and CCI classification between different groups (*P* < 0.05). By binary logistic regression analysis, we found that only CCI classification was an independent risk factor for the difference in postoperative improvement in patients with single-level lumbar discectomy, and patients with a CCI ≥ 2 were more likely to have a poor postoperative improvement rate.

## Introduction

Lumbar disc herniation (LDH) is a syndrome caused by the degeneration of the intervertebral disc, partial or total rupture of the annulus fibrosus, and compression of the nerve root and cauda equina by nucleus pulposus protrusion. It is one of the most common causes of low back and leg pain, and it is also a common degenerative disease of the spine in the clinic^1^. At least 95% of LDH occurs in L4-5 and L5-S1^2^. It has been reported that the incidence rate of LDH is approximately 2–3%; the incidence rate in males over 35 is 4.8%, and that in females is approximately 2.5%^3^. There are many causes of LDH, including disc degeneration, trauma, spinal structural abnormalities, race and heredity^2^. At present, lumbar intervertebral disc degeneration is considered the basic factor determining LDH. The intervertebral disc itself lacks blood supply. Once degenerative and injured, it is difficult to self-repair^4^.

Autophagy is a kind of cell protection mechanism found widely in eukaryotes in recent years. When the environment of cells changes (for example, due to malnutrition, hypoxia, and mechanical damage), cells can initiate the process of autophagy, form autophagosomes with double membrane structures, and fuse with lysosomes through lysosome-dependent pathways to decompose damaged organelles and long-chain proteins in the cytoplasm, which is beneficial to material recycling^5,6^. Ye W^7^ found that autophagy occurs in the degenerative intervertebral discs of rats. Ma KG^8^ found that nucleus pulposus cells can enhance their ability to degrade damaged tissues through autophagy in the face of adverse stimuli, and this autophagy process plays a very important role in the survival of nucleus pulposus cells.

In recent years, an increasing number of studies have begun to focus on the correlation between intervertebral disc autophagy intensity and disc degeneration^9^. The activity level of autophagy reflects the cell’s ability to respond to environmental stress and damage. Therefore, the autophagy intensity of the intervertebral disc may be closely related to the severity of disc degeneration and the effectiveness of postoperative recovery. If the relationship between autophagy intensity and postoperative recovery can be established, it could provide a new prognostic assessment indicator for patients and potentially offer new therapeutic targets for clinical treatment.

In addition, the Charlson Comorbidity Index (CCI), as a tool for assessing the overall health status of patients, has been widely used in recent years. Studies have shown that the CCI is closely related to patient mortality, prognosis, and treatment outcomes^10–12^. Although previous studies have confirmed the clinical significance of CCI in various diseases, its application in spinal surgery has rarely been reported. The CCI reflects the comorbidities of patients, which may significantly affect postoperative inflammation, immune responses, and the overall healing process. For example, conditions such as diabetes and cardiovascular diseases can exacerbate postoperative inflammation, inhibit tissue repair, and prolong recovery time^13,14^. Therefore, an in-depth study of how CCI impacts postoperative outcomes will not only help improve the accuracy of prognostic assessments but also provide a basis for the development of personalized treatment plans.

Based on the important role of autophagy in tissue repair and cell survival, we hypothesize that lumbar disc autophagy intensity is significantly correlated with both the degree of disc degeneration and postoperative recovery in patients. Additionally, considering that the CCI comorbidity index may affect postoperative inflammation, immune responses, and the overall healing process, we speculate that patients with a heavier comorbidity burden may experience poorer postoperative outcomes. Therefore, the purpose of this study was to explore (1) the correlation between the autophagy intensity of lumbar intervertebral discs and the degree of lumbar intervertebral disc degeneration in patients of different ages and (2) to determine whether the autophagy intensity of lumbar intervertebral discs or CCI can predict the postoperative curative effect of single-level lumbar disc herniation.

## Materials and methods

### Ethics

Ethical approval for this study was provided by the Ethics Committee of Fujian Medical University Union Hospital on March 12, 2021. The ethical review number is 2021KY028.

### Statement

We declare that we confirm that all methods were performed in accordance with relevant guidelines and regulations and that all experimental protocols have been approved by notified agencies and/or licensing committees. At the same time, we ensure that informed consent has been obtained from all subjects and/or their legal guardians.

### Study participants

This study is a retrospective study. From January 2017 to June 2019, according to age stratification (20–29, 30–39, 40–49, 50–59, 60–69, 70–79), five patients with single-level lumbar disc herniation who underwent surgical treatment in our hospital were included in each age group, and the autophagy level of the resected lumbar discs was detected by immunohistochemistry. A total of 30 patients were included and followed up for 2 years. The inclusion criteria were as follows: (1) clinical symptoms combined with imaging examination and a diagnosis of lumbar disc herniation. (2) Conservative treatment is ineffective, or the clinical symptoms are severe and require surgical treatment in our department. (3) The follow-up data and imaging examination are complete. The exclusion criteria were as follows: (1) A previous history of trauma and spinal surgery. (2) The selected patients are diagnosed with diseases that affected the function score, such as thromboangiitis obliterans, lower extremity atherosclerosis and other diseases; (3) patients complicated with an infection, tuberculosis, tumor and other diseases; and (4) patients with incomplete functional score data. Finally, according to the JOA improvement rate at the last follow-up, the patients were divided into two groups: group A (excellent-good group, JOA improvement rate > 60%) and group B (moderate-ineffective group, JOA improvement rate ≤ 60%).

### Outcome measures

The basic data included the following:

(1) Age, sex, BMI, duration of symptoms (months) and operation method.

The operation methods were divided into simple nucleus pulposus removal (-simple) and lumbar interbody fusion (-LIF).

(2) Clinical function score:

① The visual analog score (VAS) was reported on an 11-point numeric rating scale from zero (no pain) to ten (worst pain imaginable).

② The Oswestry disability index (ODI) was used to evaluate lumbar function. Pain intensity, lifting, sitting, self-care, walking, standing, sleep quality and social life were evaluated. The total score was 50 points. A higher score indicates worse lumbar function.

③ The Japanese Orthopaedic Association (JOA) scale was used to evaluate the severity and improvement of low back pain. By evaluating subjective symptoms, objective signs, daily activity restriction and bladder function, the full score was 29 points.

④ JOA improvement rate = [(JOA score after treatment – JOA score before treatment)/(29 – JOA score before treatment)] × 100%. In our study, patients were divided into the excellent-good group (Group A, JOA improvement rate > 60%, *n* = 22) and moderate-ineffective group (Group B, JOA improvement rate ≤ 60%, *n* = 8) based on the JOA improvement rate at the last follow-up.

**Fig. 1:**
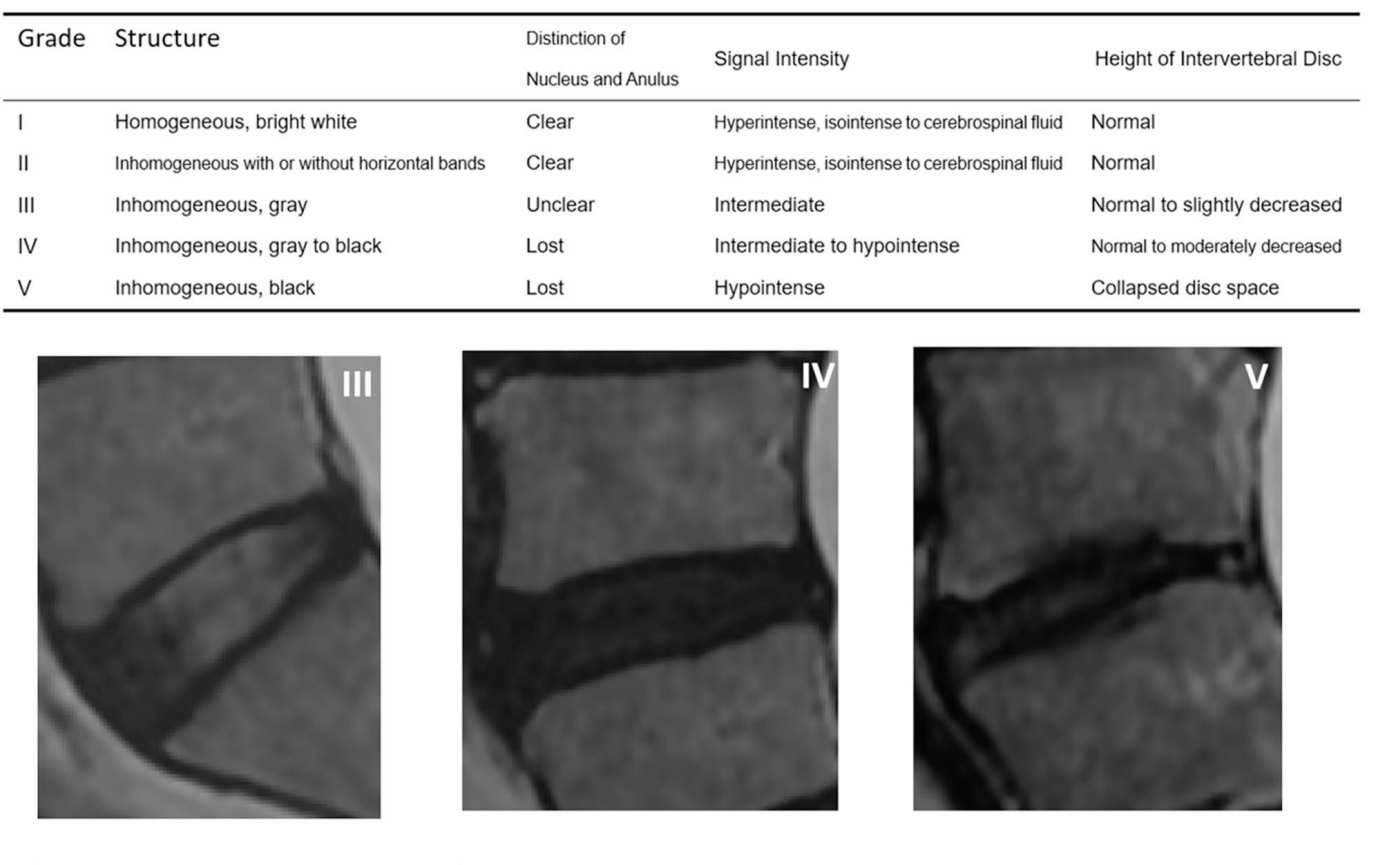
Pfirrmann classification.

(3) Imaging parameters:

① Pfirrmann classification: Lumbar disc degeneration was graded on sagittal T2-weighted MR images using the Pfirrmann classification system^15^. Each intervertebral disc from L1–2 to L5–S1 was independently evaluated by two orthopedic surgeons experienced in spinal MRI interpretation. Both reviewers were blinded to the patients’ clinical information. In cases of discrepancy, the final grade was determined through consensus discussion. The Pfirrmann grading system assesses degenerated intervertebral discs by MRI for asymmetry in disc structure, distinction of the nucleus and the annulus, signal intensity of intervertebral discs and height of intervertebral discs and assigns grades I to V for disc degeneration (Fig. 1).

**Fig. 2:**
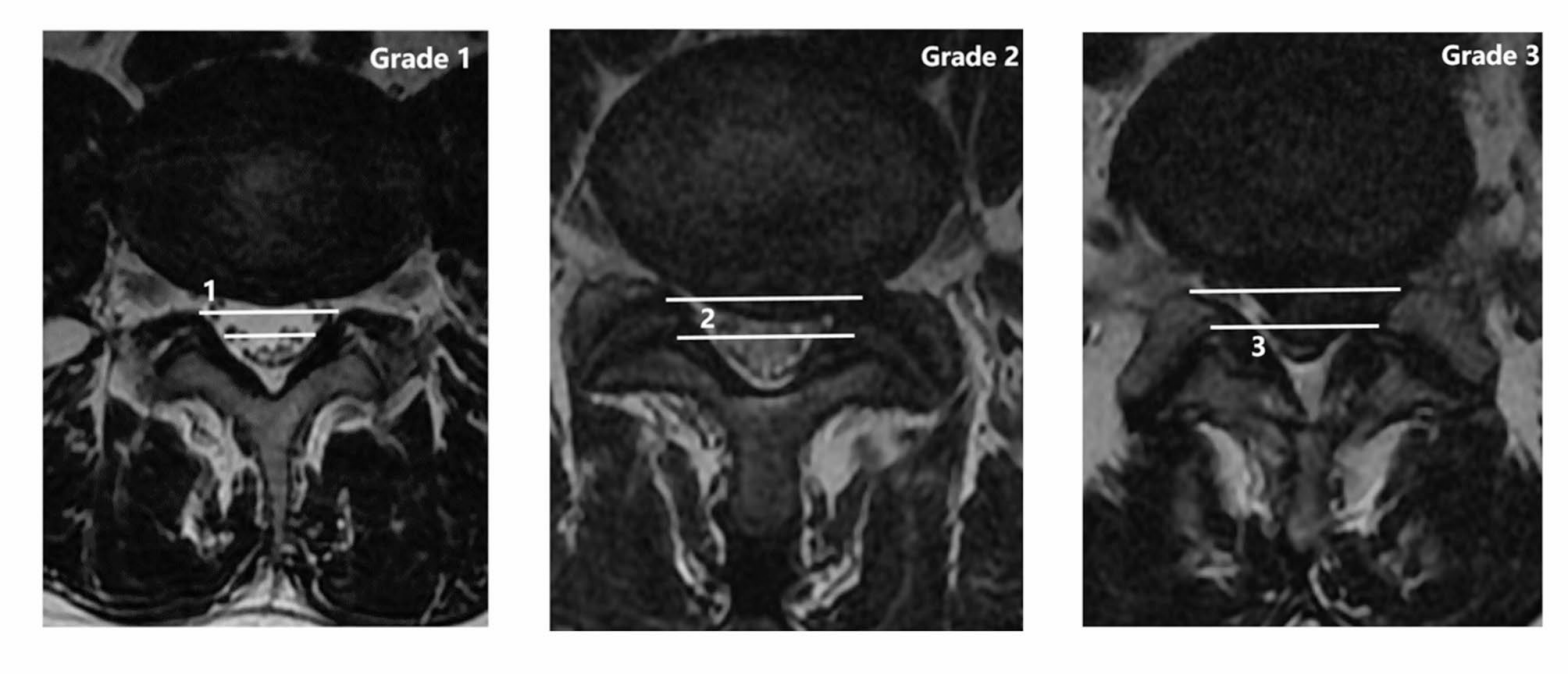
MSU classification.

② MSU classification^16^: The size and location of disc herniation are measured at the level of maximal extrusion in reference to a single intra-facet line drawn transversely across the lumbar canal and to and from the medial edges of the right and left facet joint articulations (Fig. 2). To portray the size of disc herniation, the lesion is described as 1, 2, or 3. In reference to the intra-facet line, a determination is made as to whether the disc herniation extends up to or less than 50% of the distance from the non-herniated posterior aspect of the disc to the intra-facet line (size-1) or more than 50% of that distance (size-2). If the herniation extends altogether beyond the intra-facet line, it is termed a size-3 disc. In cases of more caudal or more cephalad maximal extrusions, this measurement is taken from the posterior edge of the vertebral cortex/endplate instead of the disc. To minimize assessment bias, all imaging evaluations were performed by two independent spine surgeons who were blinded to the patients’ clinical information. In cases of disagreement, the final grade was determined through consensus discussion.

(4) Autophagy intensity and CCI classification:

① Autophagy intensity: Detection of the expression of the autophagy-specific marker protein LC3B by immunohistochemistry.

**Fig. 3:**
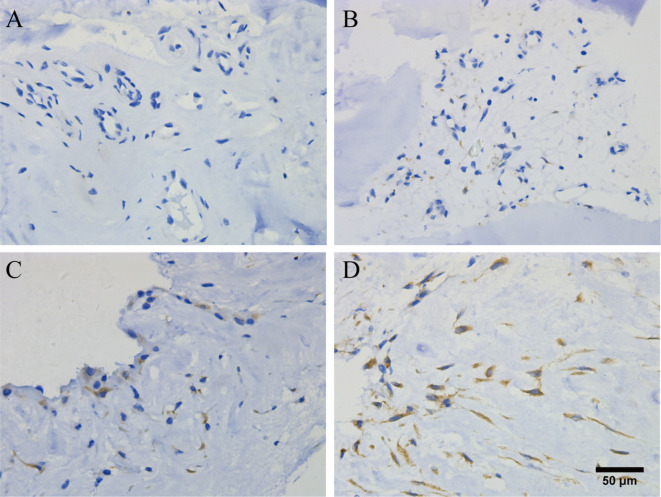
Autophagy intensity. Immunohistochemistry shows the number of positive cells expressing the autophagy marker protein LC3B. A: Basically no expression (1 point); B: Low expression (2 points); C: High expression (3 points); D: Extremely high expression (4 points).

After surgical resection, the lumbar intervertebral disc tissue was fixed with 4% formaldehyde, embedded in paraffin, and sliced continuously at 4 μm. After the sections were deparaffinized and hydrated, they were immersed in 0.01 mol/L sodium citrate buffer (pH = 6) for autoclaved antigen retrieval. After rinsing, the sections were incubated in 3% hydrogen peroxide for 15 min and then incubated with 5% bovine serum albumin blocking solution at room temperature for 15 min. The primary antibody (83506, Cell Signaling, USA) was diluted 1:150, the sections were washed and incubated with the primary antibody overnight at 4 °C, and then they were washed and incubated with the secondary antibody at room temperature for 15 min. After washing, DAB reagent was added to the slices and incubated at room temperature for 3 min. Then, the sections were counterstained with hematoxylin for 5–10 s, and after repeated soaking and washing, ethanol gradient dehydration, xylene transparency and sealing were performed. Finally, the Aperio system (Leica) was used to observe the slices, in which 5 typical areas were randomly selected to manually count the number of positive cells (400 times magnification). The results of immunohistochemical staining were evaluated by the percentage of positive cells. The positive LC3B signal was defined as brown/yellow-brown in the cytoplasm or cell membrane. For each patient, 3 typical areas were randomly selected from a slide (magnification of 400×). Score based on the proportion of positive cells: (1) 1 point, < 10% of cells stained positive; (2) 2 points, 10–25% of cells stained positive; (3) 3 points, 26–50% of cells stained positively; and (4) 4 points, > 50% of cells stained positively (Fig. 3).

To further compare the autophagy intensity, we defined 1 point and 2 points as the low expression group and 3 points and 4 points as the high expression group. All evaluations were conducted by two independent, blinded evaluators to ensure reproducibility and accuracy. In cases of disagreement, the final grade was determined through consensus discussion.

**Fig. 4:**
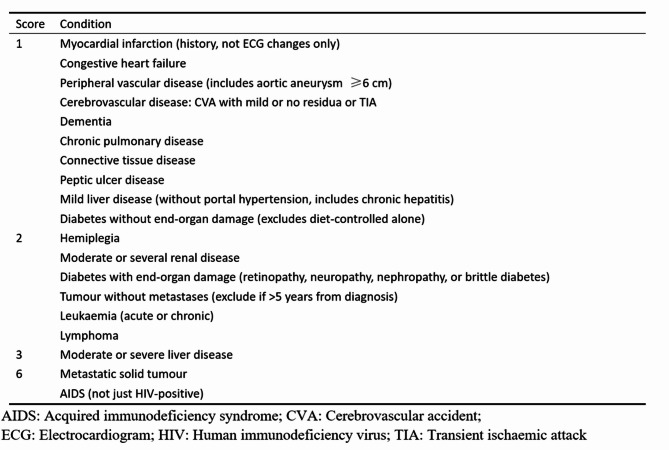
Charlson Comorbidity Index (CCI).

② CCI classification: This study used the Charlson Comorbidity Index (CCI) to assess comorbidity burden in single-level lumbar disc herniation patients. The CCI score is based on a number of conditions, including previous myocardial infarction, stroke, and liver disease, that are each assigned different weights, with a higher weight representing more severe morbidity. The summation of the weighted comorbidity scores results in a summary score (Fig. 4). For statistical analysis, patients in this study were divided into two groups according to their CCI score^12^: CCI 0–1 and CCI ≥ 2.

### Statistical analysis

SPSS 24.0 was used for statistical analyses, and statistically significant differences were identified when the P-value was < 0.05. According to age stratification (20–29, 30–39, 40–49, 50–59, 60–69, 70–79), for categorical variables, Fisher’s exact probability method and the -Wallis H test were used for intergroup analysis. For continuous variables, one-way ANOVA was used for intergroup analysis. For Group A and Group B, the intragroup analysis was performed as follows: Pearson and Spearman correlation coefficients were used to calculate the correlation between each parameter. The intergroup analysis was performed as follows: for categorical variables, Fisher’s exact probability method was used; for normally distributed continuous variables, an independent-sample t-test was used; and for nonnormally distributed variables, the Mann–Whitney U test was used. Binary logistic regression analysis was used to determine independent risk factors. To assess the presence of multicollinearity among the independent variables, variance inflation factor (VIF) analysis was performed. A tolerance value < 0.1 or a VIF > 10 was considered indicative of severe multicollinearity, while VIF values > 5 suggested moderate multicollinearity. All variables included in the logistic regression model were evaluated for VIF.

## Results

### Basic data

This study is a retrospective study. From January 2017 to June 2019, according to age stratification (20–29, 30–39, 40–49, 50–59, 60–69, 70–79), five patients with single-level lumbar disc herniation who underwent surgical treatment in our hospital were included in each age group, and the autophagy level of the resected lumbar disc was detected by immunohistochemistry. A total of 30 patients were included and followed up for 2 years. The details are described in Table 1.

**Table 1:** Demographic characteristics of the population (*n* = 30), mean functional scores and clinical classifications.

	Mean ± SD / Median(UQ ~ LQ) / *n*(%)	Range [min; max]
Age, Mean ± SD	50.3 ± 17.6	[20; 78]
Gender (Female, n (%))	14 (46.7%)	/
BMI, Mean ± SD	23.3 ± 3.6	[17.3; 34.4]
Duration of symptoms(M), Median(UQ ~ LQ)	4.0 (1.0 ~ 8.0)	[0.3; 24.0]
Pre-VAS, Mean ± SD	8.0 ± 1.4	[4.0; 10.0]
Pre-ODI, Mean ± SD	70.5 ± 16.4	[28.0; 90.0]
Pre-JOA, Mean ± SD	12.1 ± 3.3	[6.0; 20.0]
Post-VAS, Mean ± SD	1.5 ± 1.0	[0.0; 3.0]
Post-ODI, Mean ± SD	9.5 ± 8.1	[0.0; 28.0]
Post-JOA, Mean ± SD	24.4 ± 3.2	[19.0; 29.0]
JOA improvement rate (%), Mean ± SD	73.7 ± 17.8	[41.2; 100.0]
Operation method – simple – LIF	13 (43.3%) 17 (56.7%)	/ /
Autophagy intensity – high, n (%)	13 (43.3%)	/
– Low, n (%)	17 (56.7%)	/
Pfirrmann classification – III, n (%)	8 (26.7%)	/
– IV, n (%)	15 (50.0%)	/
– V, n (%)	7 (23.3%)	/
MSU classification – II, n (%)	24 (80.0%)	/
– III, n (%)	6 (20.0%)	/
CCI classification – (0–1)	18 (60.0%)	/
– (≥ 2)	12 (40.0%)	/

### Comparison among different age groups

According to age stratification, we found that there were no significant differences in the sex, BMI, duration of symptoms, MSU classification or CCI among the different age groups (*P* > 0.05). However, there were significant differences in autophagy intensity and Pfirrmann classification (*P* < 0.05), meaning that with older age, the degree of lumbar disc degeneration was more severe and the autophagy intensity was lower. The details are described in Table 2.

**Table 2:** Basic data and comparison among each group.

	Group 1 (20–29)	Group 2 (30–39)	Group 3 (40–49)	Group 4 (50–59)	Group 5 (60–69)	Group 6 (70–79)	×^2^ / F		*P*-value
Gender (Female, n (%))	2 (40%)	3 (60%)	2 (40%)	2 (40%)	3 (60%)	2 (40%)	1.071		0.957
BMI, Mean ± SD	25.3 ± 6.6	23.3 ± 2.6	25.4 ± 1.4	21.7 ± 2.7	22.7 ± 3.8	21.1 ± 1.7	1.282		0.304
Duration of symptoms(M), Median(UQ ~ LQ)	5.0 (1.8 ~ 10.0)	3.0 (2.0 ~ 24.0)	6.0 (3.2 ~ 6.0)	2.0 (0.7 ~ 8.0)	3.0 (1.5 ~ 15.0)	1.0 (0.7 ~ 8.5)	0.764		0.584
Pre-VAS, Mean ± SD	6.2 ± 1.9	7.0 ± 0.7	9.0 ± 0.7	9.0 ± 0.7	8.4 ± 0.9	8.4 ± 0.5	6.248		0.001**
Pre-ODI, Mean ± SD	47.2 ± 18.7	57.2 ± 4.1	79.6 ± 9.4	85.2 ± 3.9	78.4 ± 8.0	74.8 ± 6.9	11.327		< 0.001**
Pre-JOA, Mean ± SD	16.2 ± 4.9	14.0 ± 1.4	10.4 ± 2.5	10.2 ± 1.8	10.6 ± 2.6	11.0 ± 1.0	4.189		0.007**
Post-VAS, Median(UQ ~ LQ)	1.0 (0.0 ~ 2.0)	1.0 (0.0 ~ 1.5)	1.0 (1.0 ~ 1.5)	2.0 (2.0 ~ 2.0)	2.0 (0.5 ~ 3.0)	2.0 (1.5 ~ 3.0)	2.075		0.104
Post-ODI, Median(UQ ~ LQ)	2.0 (0.0 ~ 8.0)	4.0 (0.0 ~ 9.0)	4.0 (3.0 ~ 7.0)	16.0 (9.0 ~ 21.0)	8.0 (5.0 ~ 19.0)	14.0 (11.0 ~ 27.0)	5.032		0.003**
Post-JOA, Mean ± SD	27.4 ± 1.7	26.2 ± 3.1	25.6 ± 1.3	21.4 ± 1.7	23.4 ± 3.6	21.8 ± 3.0	4.603		0.004**
JOA improvement rate (%), Mean ± SD	88.4 ± 10.8	82.4 ± 19.0	81.6 ± 7.2	59.7 ± 7.9	68.1 ± 22.5	59.8 ± 17.0	3.359		0.019*
Autophagy intensity – high, n (%)	4 (80%)	4 (80%)	3 (60%)	2 (40%)	0 (0%)	0 (0%)	13.710		0.018*
– Low, n (%)	1 (20%)	1 (20%)	2 (40%)	3 (60%)	5 (100%)	5 (100%)			
Pfirrmann classification – III, n (%)	3 (60%)	3 (60%)	2 (40%)	0 (0%)	0 (0%)	0 (0%)	21.700		0.017*
– IV, n (%)	2 (40%)	2 (40%)	3 (60%)	4 (80%)	3 (60%)	1 (20%)			
– V, n (%)	0 (0%)	0 (0%)	0 (0%)	1 (20%)	2 (40%)	4 (80%)			
MSU classification – II, n (%)	3 (60%)	3 (60%)	5 (100%)	4 (80%)	5 (100%)	4 (80%)	5.000		0.416
– III, n (%)	2 (40%)	2 (40%)	0 (0%)	1 (20%)	0 (0%)	1 (20%)			
CCI classification – (0–1)	4 (80%)	4 (80%)	4 (80%)	2 (40%)	1 (20%)	3 (60%)	6.667		0.247
– (≥ 2)	1 (20%)	1 (20%)	1 (20%)	3 (60%)	4 (80%)	2 (40%)			

**Signify that *P* < 0.01.

*Signify that *P* < 0.05.

### Comparison between group A and group B

According to the JOA improvement rate at the last follow-up, the patients were divided into two groups: group A (excellent-good group, JOA improvement rate > 60%, *n* = 22) and group B (moderate-ineffective group, JOA improvement rate ≤ 60%, *n* = 8).

Group A (excellent-good group): A total of 22 patients, including 12 males (54.5%) and 10 females (45.5%), were included. The average age was 45.0 ± 17.1 years, and the mean BMI was 23.3 ± 3.9 kg/m^2^. The segmental distribution of lumbar lesions was as follows: L3/4 = 1 patient, L4/5 = 11 patients, and L5/S1 = 10 patients. The details are described in Table 3.

**Table 3:** Basic data and comparison between group A and group B.

	Group A (Excellent-good) (*n* = 22)	Group B (Moderate-ineffective) (*n* = 8)	T / ×^2^ / Z	*P*-value
Age, Mean ± SD	45.0 ± 17.1	64.9 ± 8.6	3.137	0.004**
Gender (Female, n (%))	10 (45.5%)	4 (50.0%)	0.049	0.825
BMI, Mean ± SD	23.3 ± 3.9	22.8 ± 2.8	-0.348	0.730
Duration of symptoms(M), Median(UQ ~ LQ)	4.0 (2.0 ~ 8.0)	1.0 (1.0 ~ 8.8)	-0.802	0.422
Pre-VAS, Mean ± SD	7.8 ± 1.5	8.5 ± 0.8	1.270	0.215
Pre-ODI, Mean ± SD	67.7 ± 17.8	78.0 ± 8.3	1.557	0.131
Pre-JOA, Mean ± SD	12.6 ± 3.7	10.9 ± 1.5	-1.835	0.077
Post-VAS, Mean ± SD	1.1 ± 0.8	2.5 ± 0.5	4.291	< 0.001**
Post-ODI, Mean ± SD	6.2 ± 5.8	18.5 ± 6.4	5.020	< 0.001**
Post-JOA, Mean ± SD	26.0 ± 2.0	19.9 ± 0.6	-12.804	< 0.001**
JOA improvement rate, Mean ± SD	82.5 ± 11.1	49.4 ± 5.3	-10.988	< 0.001**
Operation method – simple – LIF	10 (45.5%) 12 (54.5%)	3 (37.5%) 5 (62.5%)	0.151	0.697
Autophagy intensity – high, n (%)	12 (54.5%)	1 (12.5%)	4.224	0.04*
– Low, n (%)	10 (45.5%)	7 (87.5%)		
Pfirrmann classification – III, n (%)	8 (36.4%)	0 (0.0%)	6.234	0.044*
– IV, n (%)	11 (50.0%)	4 (50.0%)		
– V, n (%)	3 (13.6%)	4 (50.0%)		
MSU classification – II, n (%)	16 (72.7%)	8 (100.0%)	2.727	0.099
– III, n (%)	6 (27.3%)	0 (0.0%)		
CCI classification – (0–1)	17 (77.3%)	1 (12.5%)	10.256	0.001**
– (≥ 2)	5 (22.7%)	7 (87.5%)		

**Signify that *P* < 0.01.

*Signify that *P* < 0.05.

Group B (moderate-ineffective group): A total of 8 patients, including 4 males (50.0%) and 4 females (50.0%). The average age was 64.9 ± 8.6 years, and the mean BMI was 22.8 ± 2.8 kg/m^2^. The segmental distribution of lumbar lesions was as follows: L4/5 = 6 patients and L5/S1 = 2 patients. The details are described in Table 3.

For continuous variables, the Shapiro-Wilk normal test was used. We found that age, BMI, pre-ODI, pre-JOA, and JOA improvement rates were in line with a normal distribution. The pre-VAS, post-VAS, post-ODI, post-JOA and duration of symptoms were not in line with a normal distribution.

Through statistical analysis, we found that there was no significant difference in sex, BMI, duration of symptoms, or MSU classification between group A and group B. And there were significant differences in age, Autophagy intensity, Pfirrmann classification and CCI classification between group A and group B (*P* < 0.05). The details are described in Table 3.

By binary logistic regression analysis, we found that only CCI classification was an independent risk factor for the difference in postoperative improvement in patients with single-level lumbar discectomy, and patients with CCI ≥ 2 were more likely to have a poor postoperative improvement rate (Table 4).

**Table 4:** Using binary logistic regression analysis to judge independent risk factors.

	B	*P*-value	OR	95% Confidence Interval of OR	
				Lower Bound	Upper Bound
Age	-0.132	0.124	0.876	0.740	1.037
Autophagy intensity	1.487	0.522	4.425	0.047	419.407
Pfirrmann classification –III	/	0.885	/	/	/
–IV	17.323	0.999	/	/	/
–V	0.764	0.620	2.146	0.105	44.077
CCI classification	4.096	0.034*	60.111	1.370	2637.105

*Signify that *P* < 0.05.

To assess the potential impact of multicollinearity on the regression model, we conducted VIF analysis for the independent variables. All tolerance values were greater than 0.1 and all VIF values were below 5 (Table 5), indicating that multicollinearity was not a significant concern in the model.

**Table 5:** Multicollinearity diagnostics: tolerance and VIF for independent variables.

Variable	Unstandardized Coefficient (B)	Standard Error	Standardized Coefficient (Beta)	Tolerance	VIF
Age	-0.008	0.006	-0.325	0.431	2.320
Autophagy intensity	-0.025	0.179	-0.029	0.528	1.893
Pfirrmann classification	-0.056	0.138	-0.089	0.436	2.295
CCI classification	-0.421	0.141	-0.467	0.874	1.144

Tolerance < 0.1 or VIF > 10 indicates serious multicollinearity; VIF > 5 is considered moderate multicollinearity. All VIF values in this study were within acceptable limits, suggesting no significant multicollinearity among the variables.

### Intragroup analysis results of group A and group B

Pearson and Spearman correlation coefficients were used to calculate the correlation between each parameter. We found that in group A (excellent-good group), Autophagy intensity was negatively correlated with age and Pfirrmann classification. And in group B (Moderate-ineffective), Autophagy intensity was positively correlated with Post-JOA. The details are described in Tables 6 and 7.

**Table 6:** Correlation between each parameters in group A (excellent-good group).

	Age	Gender	BMI	Duration of symptoms(M)	Pre-VAS	Pre-ODI	Pre-JOA	Post-VAS	Post-ODI	Post-JOA	Autophagy intensity	Pfirrmann classification	MSU classification	CCI classification
Age	1													
Gender	-0.022	1												
BMI	-0.380	-0.306	1											
Duration of symptoms(M)	-0.204	0.279	0.137	1										
Pre-VAS	0.605**	0.320	-0.605**	-0.135	1									
Pre-ODI	0.676**	0.287	-0.600**	-0.225	0.949**	1								
Pre-JOA	-0.586**	-0.355	0.572**	0.062	-0.895**	-0.895**	1							
Post-VAS	0.258	-0.041	-0.455*	0.102	0.507*	0.445*	-0.449*	1						
Post-ODI	0.431*	-0.126	-0.378	0.193	0.496*	0.492*	-0.500*	0.784**	1					
Post-JOA	-0.455*	0.095	0.352	-0.219	-0.579**	-0.579**	0.546**	-0.809**	-0.849**	1				
Autophagy intensity	-0.579**	-0.083	0.354	0.121	-0.380	-0.340	0.253	-0.183	-0.197	0.331	1			
Pfirrmann classification	0.685**	-0.099	-0.459*	-0.152	0.309	0.424*	-0.324	0.224	0.347	-0.422	-0.583**	1		
MSU classification	-0.088	-0.149	0.227	-0.182	-0.247	-0.213	0.099	0.023	0.233	0.106	0.354	-0.249	1	
CCI classification	0.256	0.158	-0.265	0.186	0.226	0.270	-0.423*	0.176	0.366	-0.449*	-0.158	0.346	-0.089	1

**Signify that the correlation is significant at the 0.01 level (2-tailed).

*Signify that the correlation is significant at the 0.05 level (2-tailed).

**Table 7:** Correlation between each parameters in Group B (moderate-ineffective group).

	Age	Gender	BMI	Duration of symptoms(M)	Pre-VAS	Pre-ODI	Pre-JOA	Post-VAS	Post-ODI	Post-JOA	Autophagy intensity	Pfirrmann classification	MSU classification	CCI classification
Age	1													
Gender	-0.078	1												
BMI	-0.526	-0.399	1											
Duration of symptoms(M)	-0.007	-0.381	0.380	1										
Pre-VAS	-0.210	0.000	-0.232	-0.462	1									
Pre-ODI	-0.496	0.256	-0.149	-0.318	0.815*	1								
Pre-JOA	0.182	0.092	0.396	0.272	-0.713*	-0.775*	1							
Post-VAS	0.484	-0.500	0.181	0.403	-0.707*	-0.960**	0.642	1						
Post-ODI	0.565	-0.418	-0.377	0.255	-0.473	-0.664	0.100	0.753*	1					
Post-JOA	-0.472	0.626	0.033	-0.718*	0.442	0.481	-0.019	-0.626	-0.750*	1				
Autophagy intensity	-0.419	0.378	0.230	-0.231	0.267	0.290	0.312	-0.378	-0.664	0.709*	1			
Pfirrmann classification	0.234	0.000	-0.020	-0.120	-0.354	-0.064	-0.092	0.000	0.084	-0.209	-0.378	1		
MSU classification	/	/	/	/	/	/	/	/	/	/	/	/	1	
CCI classification	-0.525	-0.378	0.392	0.231	-0.267	-0.290	0.243	0.378	0.284	-0.079	0.143	-0.378	/	1

*Signify that the correlation is significant at the 0.01 level (2-tailed).

*Shading signify that the correlation is significant at the 0.05 level (2-tailed).

## Discussion

Lumbar disc herniation (LDH) is a common and frequently occurring disease in the clinic that seriously endangers the physical and mental health of patients. LDH has a benign natural course, and most patients with lumbar disc herniation can be improved by conservative treatment^2,17^. Therefore, conservative treatment should be the first choice for patients with LDH without significant nerve damage. The protrusion of the intervertebral disc usually atrophies with time, and the clinical function is improved. The success rate of conservative treatment is approximately 80–90%^18^, but the recurrence rate of clinical symptoms is approximately 25%^2^. The surgical methods of lumbar disc herniation can be divided into four categories: open surgery, minimally invasive surgery, lumbar fusion and lumbar artificial disc replacement. For the cases we included, we divided them into two types: ① simple nucleus pulposus removal (-simple) and ② lumbar interbody fusion (-LIF). We found that regardless of simple nucleus pulposus removal or LIF, the symptoms of patients showed good recovery, which further shows that surgery for patients with LDH is positive.

MRI is the preferred imaging examination for LDH and has the following advantages: no radiation damage, evaluation of the degeneration of the intervertebral disc, and better observation of the relationship between the herniated intervertebral disc and nerve root^19^. According to the Pfirrmann classification^15^ and MSU classification^16^, we found that the older the age, the more serious the degree of lumbar disc degeneration. Our research uses decade-based age groups, a specific categorization based on clinical experience and the typical age-related distribution patterns observed in the progression of lumbar disc degeneration. Categorizing patients into decade-based groups allows for a more systematic comparison and analysis across different age ranges, which is a commonly used approach in similar studies^20^. Meanwhile, according to the JOA improvement rate, we found that the degree of lumbar disc degeneration in the excellent-good group was lower than that in the moderate-ineffective group. However, binary logistic regression analysis showed that only CCI classification was an independent risk factor for poor postoperative improvement, which may guide us to pay more attention to the impact of the CCI on patients.

The degeneration of nucleus pulposus cells is closely related to autophagy^7,8^. Zhang TW et al.^21^ and Jin Y et al.^22^ found that autophagy can delay the degeneration of intervertebral discs, this may be related to autophagy helping to clear diseased cells, maintain normal tissue function, while reducing local inflammatory responses and improving the cellular microenvironment. In our study, we found that the older the age, the more serious the degree of lumbar disc degeneration and the lower the autophagy intensity. Meanwhile, by comparing Excellent-good Group and Moderate-ineffective Group, we found that the higher the autophagy intensity was, the better the prognosis were. This result further indicates that the degree of autophagy also plays an important role in the prognosis of patients. This finding raises important questions about how autophagy intensity might influence surgical planning for patients with LDH.

While autophagy is a critical cellular process that impacts tissue repair and regeneration, its role in predicting surgical outcomes is not yet fully understood. Based on our findings, it is possible that patients with higher autophagy intensity may have a greater potential for recovery following surgery, which could help inform the choice of surgical approach. Specifically, patients with higher autophagy levels may benefit from less invasive surgical techniques, as they may be able to heal more effectively with minimal intervention. On the other hand, patients with lower autophagy intensity may require more aggressive treatment strategies or longer postoperative rehabilitation to achieve optimal recovery. Therefore, autophagy intensity, as a potential biomarker, could complement other clinical and radiological factors to tailor surgical approaches to individual patient needs and improve overall outcomes. Further research is essential to explore the underlying mechanisms of autophagy in the context of LDH and to evaluate its role in guiding clinical decisions, particularly regarding the selection of surgical techniques and postoperative care strategies.

In our study, while there is a significant correlation between autophagy intensity and lumbar intervertebral disc degeneration, we also recognize that confounding factors such as variability in surgical techniques, rehabilitation protocols, and patient lifestyle factors could have a significant impact on postoperative recovery and long-term prognosis. For example, different surgical approaches (such as minimally invasive surgery versus traditional surgery) and rehabilitation protocols may result in variations in postoperative recovery and outcomes^23,24^. Additionally, lifestyle factors such as activity level, diet, smoking, and alcohol consumption may also have a notable effect on surgical outcomes^25–27^. These factors were not fully controlled in our study, and therefore, future research should take these confounding variables into account and further explore the independent role of autophagy intensity in predicting surgical outcomes.

However, while autophagy has been shown to impact disc degeneration, its relationship with inflammatory cytokines, which are critical mediators in both disc degeneration and postoperative recovery, remains underexplored. Inflammatory cytokines such as TNF-α, IL-1β, and IL-6 are known to promote disc degeneration by enhancing matrix degradation and inflammatory responses. Additionally, these cytokines play significant roles in impairing tissue repair mechanisms, which can delay postoperative recovery^28,29^. Autophagy can help control inflammation by clearing damaged cellular components and reducing inflammasome activation^30^. This modulation of inflammation not only limits tissue damage but also supports tissue homeostasis, which is critical for disc regeneration and healing after surgery. Therefore, the interplay between autophagy and inflammatory cytokines may be a key factor in both the progression of disc degeneration and the postoperative recovery process.

Since the Charlson Comorbidity Index (CCI) was proposed, an increasing number of studies have shown that it has a significant correlation with mortality, prognosis and curative effect^10–12^. Sim DS et al.^12^ grouped patients based on CCI scores (CCI 0–1 and CCI ≥ 2) to assess the comorbidity burden in hip fracture patients. However, there are few studies on the CCI in spinal surgery. In our study, we found that only CCI classification was an independent risk factor for the difference in postoperative improvement in patients with single-level lumbar discectomy, and patients with CCI ≥ 2 were more likely to have a poor postoperative improvement rate. Therefore, the CCI may warrant greater attention in clinical practice, as it could potentially be associated with postoperative recovery outcomes.

This study is based on patients with single-level LDH to research the risk factors for postoperative curative effects. We found that the older the age was, the more serious the degree of lumbar disc degeneration and the lower the autophagy intensity. For patients with single-level lumbar disc herniation, the higher the autophagy intensity was, the better the prognosis were. And CCI classification was an independent risk factor for predicting postoperative efficacy. When the CCI ≥ 2, the postoperative prognosis is more likely to be poor. Furthermore, the relationship between autophagy and inflammatory cytokines may offer further insights into the mechanisms underlying both disc degeneration and postoperative recovery.

However, our research also has several shortcomings and issues that need to be further explored. To begin with, due to its retrospective nature, there is potential for selection bias and recall bias, as patients’ medical history and symptom recollection may not be fully accurate. We tried to minimize these biases by using clinical records and imaging data, but they cannot be completely eliminated. Moreover, the study was conducted at a single institution, lacking external validation, which limits the generalizability of the findings. Future multi-center studies are needed to validate our results. In addition, the sample size was small (30 patients), which may not be sufficient to draw comprehensive conclusions. However, the primary aim of this study was to conduct a preliminary exploratory analysis to identify potential trends and biological significance, and larger studies are needed to confirm our findings. Confounding factors such as variations in surgical techniques, rehabilitation protocols, and lifestyle were also not controlled for in this study and should be considered in future research. Furthermore, although LC3B immunohistochemistry was used to evaluate autophagy levels and is widely adopted in autophagy-related studies, it only reflects autophagosome accumulation and cannot distinguish between increased autophagy initiation and impaired autophagic flux. As a result, our assessment represents only a static snapshot of autophagy and does not fully capture its dynamic process. Future studies should consider incorporating complementary methods, such as p62/SQSTM1 or electron microscopy, to achieve a more accurate and comprehensive evaluation. Additionally, there may be overlapping variance among variables such as age, Pfirrmann classification, and autophagy intensity, potentially leading to multicollinearity in the regression analysis. To assess this issue, we performed VIF analysis on all variables included in the model. All VIF values were below the commonly accepted threshold (VIF < 5), indicating that multicollinearity was not a significant concern. Nevertheless, some degree of variance overlap may still influence the stability and interpretability of individual regression coefficients. In conclusion, while this study provides valuable preliminary insights, further research is necessary to confirm these results and enhance their applicability.

## Conclusion

The degree of degeneration and autophagy intensity of lumbar intervertebral discs were significantly correlated with age. The older the age was, the more serious the degree of lumbar disc degeneration and the lower the autophagy intensity. For patients with single-level lumbar disc herniation, CCI classification was an independent risk factor for predicting postoperative efficacy. When CCI ≥ 2, the postoperative prognosis was more likely to be poor. Therefore, the CCI may warrant greater attention in clinical practice, as it could potentially be associated with postoperative recovery outcomes.

## Data Availability

The data that support the findings of this study are available from the corresponding author upon reasonable request.
